# Molecular screening of tsetse flies and cattle reveal different *Trypanosoma* species including *T. grayi* and *T. theileri* in northern Cameroon

**DOI:** 10.1186/s13071-017-2540-7

**Published:** 2017-12-29

**Authors:** Sen Claudine Henriette Ngomtcho, Judith Sophie Weber, Elisabeth Ngo Bum, Thaddeus Terlumun Gbem, Sørge Kelm, Mbunkah Daniel Achukwi

**Affiliations:** 1 Department of Biological Sciences, University Ngaoundéré, P.O. Box 454, Ngaoundéré, Cameroon; 2 Ministry of Public Health, Regional Hospital of Ngaoundéré, Ngaoundéré, Cameroon; 3 Centre for Biomolecular Interactions Bremen, Department of Biology and Chemistry, University Bremen, 28334 Bremen, Germany; 4 Africa Centre of Excellence for Neglected Tropical Diseases and Forensic Biotechnology, Ahmadu Bello University, Zaria, Nigeria; 5 Department of Biology, Ahmadu Bello University, Zaria, Nigeria; 6 TOZARD Research Laboratory, P.O. Box 59, Bambili-Tubah, Bamenda, Cameroon

**Keywords:** *Trypanosoma grayi*, *Trypanosoma theileri*, Bodonidae, Cattle, Tsetse fly, ITS1, Trypanosomosis, Northern Cameroon

## Abstract

**Background:**

African trypanosomes are mainly transmitted through the bite of tsetse flies (*Glossina* spp.). The present study investigated the occurrence of pathogenic trypanosomes in tsetse flies and cattle in tsetse fly-infested areas of Northern Cameroon.

**Results:**

Trypanosomes were identified using nested polymerase chain reaction (PCR) analysis of internal transcribed spacer 1 (ITS1) region, both by size estimation and sequencing of PCR products. Apparent density indices recorded in Gamba and Dodeo were 3.1 and 3.6 tsetse flies per trap and day, respectively. *Trypanosoma* prevalence infection rate for the tsetse fly gut (40%) and proboscis (19%) were recorded. Among the flies where trypanosomes were detected in the gut, 41.7% were positive for *T. congolense* and 14.6% for *T. brucei* ssp., whereas in the proboscis 36% harboured *T. congolense* and 62% contained *T. vivax*. *T. grayi* was highly prevalent in tsetse fly gut (58%). The most common mixed infections were the combination of *T. congolense* and *T. grayi*. Trypanosome prevalence rate in cattle blood was 6%. Among these, *T. vivax* represented 26%, *T. congolense* 35%, *T. brucei* ssp. 17% and *T. theileri* 17% of the infections. Surprisingly, in one case *T. grayi* was found in cattle. The mean packed cell volume (PCV) of cattle positive for trypanosomes was significantly lower (24.1 ± 5.6%; *P* < 0.05) than that of cattle in which trypanosomes were not detected (27.1 ± 4.9%). Interestingly, the occurrence of *T. theileri* or *T. grayi* DNA in cattle also correlated with low PCV at pathological levels.

**Conclusion:**

This molecular epidemiological study of *Trypanosoma* species in Northern Cameroon revealed active foci of trypanosomes in Dodeo and Gamba. These findings are relevant in assessing the status of trypanosomosis in these regions and will serve as a guide for setting the priorities of the government in the control of the disease.

**Electronic supplementary material:**

The online version of this article (10.1186/s13071-017-2540-7) contains supplementary material, which is available to authorized users.

## Background

Trypanosomes are a group of flagellated protozoans that include *Trypanosoma brucei gambiense*, responsible for human African trypanosomiasis (HAT) in West and Central Africa, and *Trypanosoma brucei rhodesiense* in East Africa [1]. Several other species are responsible for animal African trypanosomosis (AAT), also called “nagana”, mostly caused by *T. congolense*, *T. vivax* and *T. brucei brucei* [2]. These salivarian parasites are transmitted during the blood meal of an infected vector insect, whereas stercorarian trypanosomes, i.e. *T. grayi*, develop in the gut and are transmitted via faeces [3].

Usually, tsetse flies (Diptera: Glossinidae) are vectors of pathogenic trypanosomes in sub-Saharan Africa [4]. However, other biting flies like the Tabanidae and species of *Stomoxys* can mechanically transmit parasites [5, 6]. Thirty-three extant species and sub-species of *Glossina*, restricted to sub-Saharan Africa, have been reported [7]. They have been divided into three groups based on their distribution patterns and morphological features and include the savannah species (e.g. *G. morsitans*), the forest species (e.g. *G. fusca*) and the riverine species (e.g. *G. palpalis*). In the vector *Glossina*, *T. congolense* colonises the gut and the proboscis, whereas *T. brucei* spp. colonise the gut and the salivary glands [8]. *Trypanosoma vivax* occurs exclusively in the proboscis and degenerates when ingested with a blood meal to the mid-gut [9]. As transmission depends wholly on feeding, the trypanosomosis risk is usually related to tsetse fly density, trypanosome infection rates, and contact between hosts and vectors [1]. Remote rural areas are typically prone to high levels of the disease. Considered the “beef basket” region of Cameroon, several control strategies were carried out in the Adamawa region targeting bovine trypanosomosis to mitigate its devastating impact on livestock [10]. However, its recent resurgence has been favoured by factors such as climate and vegetation changes, and the interruption of control and treatment programs [11]. The stoppage of tsetse fly control activities by aerial spraying of insecticides in the Adamawa region [12] has led to an exaggerated threat from tsetse flies, which occur towards the north of the area [11]. Trypanosomosis is devastating and imposes major economic constraints on livestock farmers, least capable of affording treatment costs. To establish successful control measures, it is essential to identify the different *Trypanosoma* species in circulation and evaluate their distribution in vector and mammalian hosts. While some information exists about the trypanosomiasis status in South Cameroon [13–16], little data is available for the northern area of the country, especially regarding molecular epidemiology. The main information available is that areas previously free of tsetse flies have been reinvaded [11], despite the action of the Mission spéciale d’éradication des glossines (MSEG), which was established to eradicate tsetse flies. Several studies limited to parasitological and serological parameters of trypanosomosis have been undertaken in this region [12, 17–20]. DNA-based approaches for the detection of trypanosomes, such as those using polymerase chain reaction (PCR) [21–24], have greatly improved in the last decades. Taking advantage of this, we used PCR-based methods to identify different *Trypanosoma* species in the gut and proboscis tissue from tsetse flies as well as in blood samples from cattle in some villages in the Adamawa and the northern region of Cameroon. Obtained results will contribute to developing guidelines for trypanosomosis control measures in this area.

## Methods

### Study sites

**Fig. 1:**
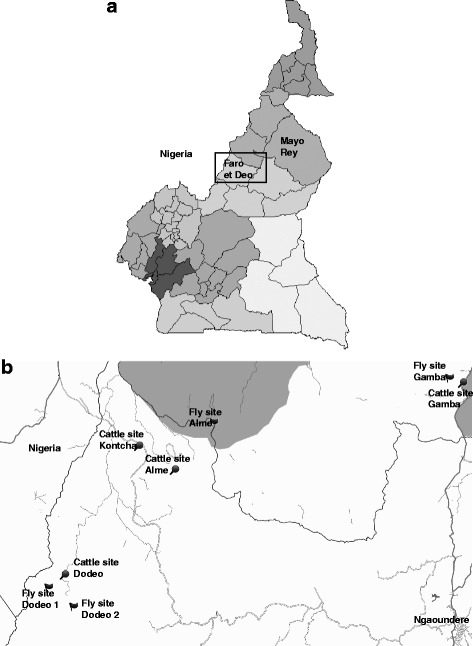
Maps of the study areas. **a** The study area lies in the north of Cameroon, showing the administrative divisions. **b** Site locations: Gamba in the North Region and Dodeo, Alme and Kontcha in the Adamawa Region. Sites for flies trapping and cattle sampling are indicated. Grey shaded areas indicate the national parks Faro (near Alme) and Bénoué (near Gamba)

This study was undertaken in Northern Cameroon, in the vicinity of Adamawa and the North Region. During the dry season between November and April the dry wind comes from the northeast, originating in the Sahara Desert. From May to October, which is the wet season, humid air is drawn from the southwest. Adamawa lies at 6°20′N, 13°30′E and covers an area of 63,701 km^2^ (Fig. 1). It is dominated by sparse tree vegetation known as Guinea savannah [10]. The survey was conducted in the Faro et Deo division (Fig. 1), which covers an area of 10,435 km^2^ with a total population of about 67,000 in 2005 [25]. The North Region lies at 8°30′N, 14°00′E and covers an area of 66,090 km^2^. The target division in the North Region was Mayo-Rey (Fig. 1), which covers an area of 36,529 km^2^ with a total population of about 375,000 in 2005 [25].

### Tsetse fly collection and processing

A cross-sectional tsetse fly survey was conducted along the main rivers of both Adamawa (Mayo Deo) and the North Region (Bénoué) of Cameroon during the dry season in March 2014. In Dodeo, flies were collected at two separate sites (Fig. 1). At both sites, traps were placed along small rivers. The forest gallery vegetation along the river banks provided shadows leading to a relatively cool environment. In Gamba and Alme, the collection sites were characterised by a savannah gallery. Twelve biconical traps and four Vavoua traps were set up at each site with 100 m spacing and baited with acetone. To keep as many flies as possible alive in the traps, these were placed as much as possible in the shade of trees. Traps were deployed around 7:00 h and left for 2 days. The traps were inspected twice every day for fly collection at 12:00 h and 17:00 h. In areas that were very remote and difficult to access, the harvest took place once per day, around 17:00 h. The number of flies per trap and time of collection were recorded. To assess the relative abundance of tsetse flies at each trapping site, the apparent density was estimated as the total number of tsetse flies caught per trap per day [26]. Geographical coordinates were documented using a GPS device (Garmin GPSMAP 60csx) and environmental parameters (temperature and relative humidity) recorded using a data logger (EasyLog TH, Lascar, Whiteparish, UK).

Tsetse flies were identified using morphological characteristics such as a distinct proboscis, folded wings at rest with hatchet cells, and branched arista hairs on the antennae. Males have a hypopogium and are smaller than females which have a vulva. The following key characteristics were used to identify *Glossina* species: the colour of the tarsal segments of the hind and front legs, and the colour and the shape of the dorsal surfaces of the abdomen with or without banding [27]. Identification of tsetse fly species was further confirmed by sequencing parts of the cytochrome *c* oxidase 1 (*cox*1) [28] as described below.

Gut and proboscis tissues were dissected from each fly and placed in a 1.5 mL cryotube, containing 200 μL of nucleic acid preservation agent (NAPA; 25 mM sodium citrate, 10 mM ethylenediaminetetraacetic acid (EDTA), 70 g ammonium sulfate/100 ml solution, pH 7.5). Samples were kept at -20 °C in the field, and subsequently transferred to -80 °C upon arrival in the laboratory. Non-teneral flies were recorded. To avoid contamination between tissues, the proboscis was removed from the flies’ head before opening the abdomen. To avoid cross-contamination, we used fresh dissection pins and forceps for each fly tissue, decontaminated by incubation in 5% sodium hypochlorite solution for 20 min and subsequently washed thoroughly with double distilled water.

### Blood collection from cattle, processing and PCV measurement

Blood was collected from the jugular vein of 392 cattle directly into blood collection tubes containing EDTA (Sarstedt, Nürnbrecht, Germany). The collected blood was centrifuged at 3000× *rpm* for 15 min. The upper layer containing plasma and the buffy coat was collected separately in 1.5 mL cryotubes. NAPA was added to the buffy coat resulting in a 1:4 final dilution. The PCV of each blood sample was measured following centrifugation in heparinised haematocrit capillary tubes at 12,000× *rpm* for 5 min (haematocrit centrifuge from Hawksley, UK). An animal with a PCV value below 25% was considered to be anaemic [11].

### DNA extraction and quantification

DNA was extracted from tsetse fly gut or buffy coat from cattle blood using the DNeasy Blood and Tissue Kit (Qiagen, Hilden, Germany) according to the manufacturer instructions and photometrically quantified using Nanodrop 1000 apparatus (Thermo Scientific, Dreieich, Germany) at a wavelength of 260 nm. To obtain DNA from proboscis, they were ground using a single-use microfuge pestle (Sigma Aldrich, Munich, Germany) in a 1.5. ml microfuge and taken up in 50 μl PBS. The homogenised tissue was then used for PCR without further DNA purification.

### Identification of *Glossina* species by sequencing of the *cox*1 gene

To identify tsetse fly species also on the genetic level, we amplified and sequenced part of the *cox*1 gene [28] using the primers listed in Table 1. PCR reactions (25 μl) contained 5 μl template DNA, 2 μM of primers, 20 μM of dNTPs (Thermo Fisher Scientific, Dreieich, Germany) and Dream*Taq* Green polymerase (Thermo Fisher Scientific). PCR cycling reactions included an initial denaturation 95 °C for 5 min, followed by 35 cycles of 1 min at 94 °C, 1 min at 55 °C, and 2 min at 72 °C, and a final elongation of 10 min at 72 °C. Fragments were purified on a 1.5% agarose gel containing 0.5 μg/ml of SERVA DNA Stain G (SERVA, Heidelberg, Germany) and sequenced as described below.

**Table 1:** Primers used in this study to amplify different *Trypanosoma* species

Name	Sequence (5′–3′)	TA (°C)	Amplicon size (bp)	Species	Reference
ITS1-OutF	TGCAATTATTGGTCGCGC	54	Variable	All *Trypanosoma* species	[22]
ITS1-OutR	CTTTGCTGCGTTCTT				
ITS1-InF	TAGAGGAAGCAAAAG				
ITS1-InR	AAGCCAAGTCATCCATCG				
TCON-OutF	TGCAATTATTGGTCGCGC	54	681 (kilifi) or 781 (forest)	*T. congolense*	[22]
TCON-OutR	TGCAATTATTGGTCGCGC				
TCON-InF	TCGCGTGTCTCACGT				
TCON-InR	TCAAAGATTGGGCAATGT				
TGR-OutF	TGGCAGACACATACCTGCCA	54	526	*T. grayi*	This study
TGR-OutR	TGGGGATTACGGATGAAAC				
TGR-InF	TTAAGGAGGCGCTCAGGTTC				
TGR-InR	TGTGCATATACGTCTATG				
TVIV-F	CTGAGTGCTCCATGTCCCAC	60	142	*T. vivax*	[24]
TVIV-R	CCACCAGAACACCAACCTGA				
COI-F	TTGATTTTTTGGTCATCCAGAAGT	55	900	Generic *Glossina cox*1	[28]
COI-R	TGAAGCTTAAATTCATTGCACTAATC				

*Abbreviations*: TCON, *T. congolense*; TGR, *T. grayi*; TVIV, *T. vivax*; *cox*1, cytochrome *c* oxidase 1; Out, outer primer; In, inner primer; F, forward; R, reverse; TA, annealing temperature

### Detection and identification of *Trypanosoma* species

Nested PCR targeting the internal transcribed spacer 1 (ITS1) region of the trypanosome ribosomal DNA, which separates 28S from 5.8S RNA, was performed. First, identification was made using size estimation of amplicons generated by use of generic primers (Table 1). For further confirmation of the species, primer sets specific for several *Trypanosoma* species were designed to amplify regions of trypanosomal 18S ribosomal RNA (Table 1).

ITS1 nested PCR reactions for detection of trypanosomal DNA with generic primers were performed in a 25 μl reaction volume containing Dream*Taq* Green DNA polymerase and Dream*Taq* Green buffer (Thermo Scientific). The first reaction containing 1 ng/μl of DNA template and 2 μM of primers (ITS1-OutF and ITS1-OutR, Table 1) was run under the following conditions: initial denaturation at 95 °C for 1 min, 30 cycles of 94 °C for 1 min, annealing at 54 °C for 30 s, elongation at 72 °C for 30 s, followed by a final elongation step at 72 °C for 5 min. First PCR products were diluted 80-fold and 1 μl of this dilution was used for the second PCR reaction with ITS1-InF and ITS1-InR primers (Table 1) under the same conditions as the first reaction. As discussed by Adams et al. [24], with this nested PCR, DNA of a single parasite can be detected.

Regarding specific identification, the annealing temperature was 54 °C for the first reaction and was varied during the second reaction based on the melting temperatures of the primer sets 60 °C for *T. vivax* and 54 °C for all other *Trypanosoma* species. To optimise the PCR with primers specific for *T. grayi*, the MgCl_2_ concentration of the Dream*Taq* Green buffer (Thermo Scientific) was increased by adding 2 mM MgCl_2_. Amplified products were resolved by electrophoresis on 1.5% or 2% agarose gels.

### Purification and subcloning of selected PCR products

Selected PCR products were carefully excised from the gel using a clean scalpel. DNA was purified using GeneJet Gel Extraction Kit (Thermo Scientific), following the instructions of the manufacturer. DNA concentrations were determined at a wavelength of 260 nm on a Nanodrop 1000 apparatus (Thermo Scientific). Purified PCR products were cloned into either the linearized plasmid vector PCR™ 2.1-TOPO (Thermo Scientific) with single 3′- deoxythymidine (T) overhangs or linearised pJET 1.2/blunt plasmid using the CloneJET PCR (Thermo Scientific), according to the manufacturer’s instructions. Positive clones were identified by colony PCR and selected single colonies were cultured in LB plus ampicillin (100 μg/ml) with shaking overnight at 37 °C. Bacteria were collected by centrifugation (4500× *g*, 15 min at 4 °C) and the plasmid DNA was purified using the NucleoBond Xtra Midi Plus MidiPrep Kit (Macherey-Nagel, Düren, Germany), or GeneJET Plasmid MiniPrep Kit (Thermo Fischer Scientific), according to the instructions of the manufacturer.

### Sequencing of PCR products

In the initial phase of the project, subcloned PCR products were sequenced employing the Big Dye Terminator v3.1 Cycle Sequencing Kit (Applied Biosystems, Dreieich, Germany) according to the manufacturer’s instructions. The samples were incubated for 2 min at 95 °C for initial denaturation of DNA, followed by 60 sequencing reaction cycles (95 °C for 15 s, 58 °C for 15 s, 60 °C for 4 min and 60 °C for 7 min) and then held at 4 °C. Reactions were then purified using Sephadex 50 in 96 well plates previously equilibrated with distilled water at 4 °C and sequencing was done at the Max Planck Institute for Marine Microbiology in Bremen, using a Genetic Analyzer (Applied Biosystems).

In a later stage of the project, PCR products were sequenced directly after gel purification or following subcloning into a pJET1.2/blunt vector (Thermo Scientific) by SeqLab, Göttingen, Germany.

### Bioinformatics and statistical analysis

Obtained data were evaluated using SPSS software version 22.0. Chi-square analysis was employed to compare prevalence rates. A Student’s t-test (unpaired, two-tailed) was used to compare mean PCV values. Differences were tested for significance at *P* < 0.05. Geneious bioinformatics software (Biomatters, Auckland, New Zealand) was used to analyse the sequencing results. For alignments of the DNA sequences the matrix of the Geneious Alignment tool was used, applying a gap open penalty of 12, a gap extension penalty of 1.5 and the alignment type “global alignment with free end gaps”. The cost matrix was set to 51–93% alignment with free end gaps, depending on the degree of similarity. Sequences were screened against databases using nucleotide BLAST searches (Megablast) at NCBI website (http://blast.ncbi.nlm.nih.gov/Blast.cgi) or TriTrypDB (version 6.0; http://tritrypdb.org) against the whole genome database.

## Results

### *Glossina* species in the study area

A total of 241 tsetse flies were trapped at the two study sites over a 2-day period (Table 2). Density indices of tsetse flies were 3.1 in Gamba and 3.6 in Dodeo indicating similar tsetse fly population densities in these areas. In Alme, only a single tsetse fly was caught. In Dodeo, *G. palpalis palpalis* and a *Glossina* sp. not identified by *cox*1 sequencing were collected, whereas in Gamba *G. tachinoides* and *G. morsitans submorsitans* were recorded. Overall, the main tsetse species identified were *G. p. palpalis* and *G. m. submorsitans* (Table 2, Additional file 1: Tables S1-S4).

**Table 2:** The numbers and species of *Glossina* sampled from each study area in northern Cameroon. *Glossina* species were identified using morphology and by sequence analysis of the cytochrome *c* oxidase 1 gene

Study sites	*Glossina palpalis palpalis*	*Glossina morsitans submorsitans*	*Glossina tachinoides*	*Glossina* sp.	Total
Dodeo	109	0	0	18	127
Alme	0	1	0	0	1
Gamba	0	103	10	0	113
Total	109	104	10	18	241

### *Trypanosoma* species identified

**Fig. 2:**
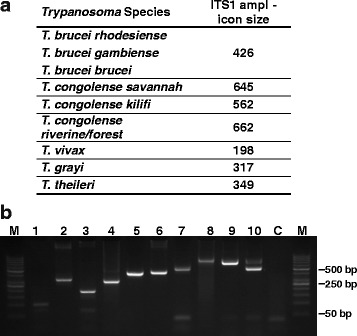
ITS1 amplicons of *Trypanosoma* species. **a** Amplicon sizes expected for amplification with generic primers ITS1-InF and ITS1-InR were calculated from available database sequences and crosschecked with sequences from screened samples. **b** PCR was performed with representative samples containing DNA of the indicated origin and generic (**a**) or specific primers (Table 1). Lane M: Marker GeneRuler 50 bp Ladder (Thermo Scientific); Lane 1: NI100 (generic primers); Lane 2: Bodonid (generic primers); Lane 3: *T. vivax* (generic primers); Lane 4: *T. grayi* (generic primers); Lane 5: *T. theileri* (generic primers); Lane 6: *T. brucei* ssp. (generic primers); Lane 7: *T. grayi* (specific primers); Lane 8: *T. congolense* (specific primers); Lane 9: *T. congolense* forest (specific primers); Lane 10: *T. congolense* forest and *T. congolense* kilifi (specific primers); Lane C: control without DNA template

The identification of trypanosomes was performed by PCR amplification of ITS1. Initially, trypanosome species were preliminarily assigned according to the band size of the product yielded. As described in the literature [24], sizes in the range between 550 and 700 bp, 400–450 bp and 198–250 bp were considered to come from *T. congolense* kilifi and *T. congolense* forest/savannah, *T. brucei* ssp., and *T. vivax*, respectively. Also, unexpected band sizes of about 320 and 100 bp were amplified (Fig. 2b, Additional file 1: Tables S1-S3).

To further check the identification, samples were subjected to PCR with specific primers where available. Finally, representative PCR products were subcloned and sequenced for final confirmation or more detailed identification. The fragment sizes and respective *Trypanosoma* species are listed in Fig. 2 and Additional file 1: Tables S1-S4).

### Identification of non-assigned amplicons

**Fig. 3:**
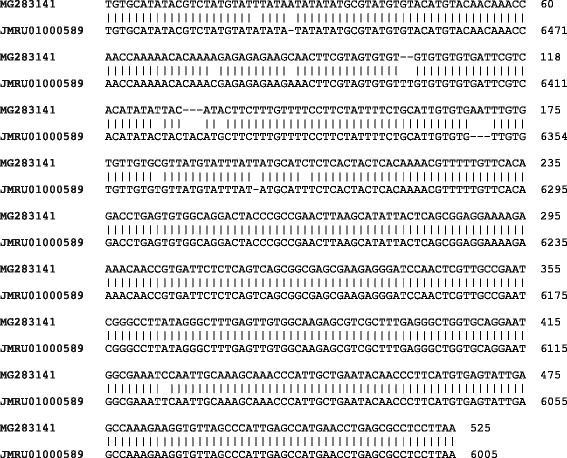
The sequence of an amplicon obtained from tsetse fly gut with primers specific for *T. grayi*. Specific primers (TGR-In primer set) targeted against *T. grayi* amplified a 525 bp fragment (MG234546, Additional file 1: Table S4) from tsetse fly gut sample (ID 237-51-00211-1-40-10, *G. tachinoides*, Additional file 1: Table S4). The fragment was sequenced and aligned with the corresponding fragment of genomic DNA from *T. grayi* ANR4 (JMRU01000589)

To identify the species yielding the amplicons of about 320 bp, several independent non-identified PCR products obtained from sampled tsetse tissues were purified, subcloned and sequenced. The results revealed that these amplicons represent DNA products of 310 to 317 bp, which are 91% identical to *T. grayi* ANR4, (Additional file 2: Figure S1). Most of the differences were small insertions or deletions in the range of 1 to 10 nucleotides. To generate a tool for species identification of possible *T. grayi* candidates, specific primers annealing in the 18S rRNA gene were designed based on the genomic sequence of *T. grayi* ANR4 (JMRU01000589). Primers were tested against genomic DNA from *T. grayi* ANR4 (generously provided by W. Gibson). With these primers, PCR products ranging in size between 520 and 530 bp were amplified from corresponding tsetse fly tissues samples. Sequencing the amplicons gave 97 to 99% sequence identity to *T. grayi* ANR4 (Fig. 3).

Five cattle blood samples presented similar PCR products of about 320 bp (Additional file 1: Table S3). Interestingly, in one of these cases, the sequence was also 98% identical to *T. grayi* ANR4, thus revealing the presence of *T. grayi* in cattle. However, the sequences of the other four 320 bp PCR products obtained from cattle samples revealed *T. theileri* as the parasite (Table 3). ITS1 sequences of *T. theileri* show a relatively high degree of diversity (65–100% sequence identity) [29, 30]. Alignment of the sequences obtained in this study indicated that the samples were derived from two distinct strains, which were also found in the database (Table 3). Within each strain, the sequences were 95–98% identical, whereas sequence identity between these strains was only about 70%.

**Table 3:** Percentage sequence identity matrix comparing *T. grayi* and *T. theileri* sequences from cattle blood samples

		Cattle				*T. theileri*			
	*T. grayi* ANR4	4^a^	15^a^	165^a^	361^a^	Tthc29^b^	SitaBip1^b^	Cow isolate^b^	Tthb10^b^ (HQ664808)
Cattle 321^a^ (*T. grayi*, MG255205)	98	63	62	54	53	47	47	60	53
*T. grayi* ANR4 (JMRU01000589)		64	63	54	53	47	48	61	54
Cattle 4^a^ (*T. theileri*, MG255206)			97	67	67	62	62	91	77
Cattle 15^a^ (*T. theileri*, MG255207)				67	67	62	62	89	75
Cattle 165^a^ (*T. theileri*, MG255208)					99	97	82	66	56
Cattle 361^a^ (*T. theileri*, MG283143)						98	82	65	56
*T. theileri* Tthc29^b^(HQ664818)							83	61	57
*T. theileri* SitaBip1^b^ (HQ664843)								61	60
*T. theileri* cow isolate^b^ (JX853185)									82

^a^Cattle 4 and 15 were from the same herd sampled in Dodeo, cattle 165 was sampled in Alme, cattle 321 in Kontcha and cattle 361 in Gamba

^b^ *T. theileri* Tthc29 and *T. theileri* Tthc10 were isolated from cattle in Thailand [29]. *T. theileri* SitaBip1 was isolated from marsh buck in south Cameroon [61], identified as *T. theileri* [45] and the ITS1 sequence published by Garcia et al. [29]. *T. theileri c*ow isolate was isolated from a cow in the USA [60]

The same approach as employed for the 320 bp PCR products was undertaken to identify the origin of 100 bp amplicons. However, it was observed that the sequences of subcloned PCR products were heterogeneous. Whereas several sequences looked like primer artefacts, in several samples sequence fragments were identified, which are highly conserved in the Kinetoplastidae providing a hint towards the possible kinetoplastid origin. Therefore, the 100 bp PCR products are further referred to as NI100 (non-identified 100 bp PCR product), and the corresponding samples were not included as *Trypanosoma*-positive in the calculation of prevalence rates.

### *Trypanosoma* species in tsetse fly gut

**Fig. 4:**
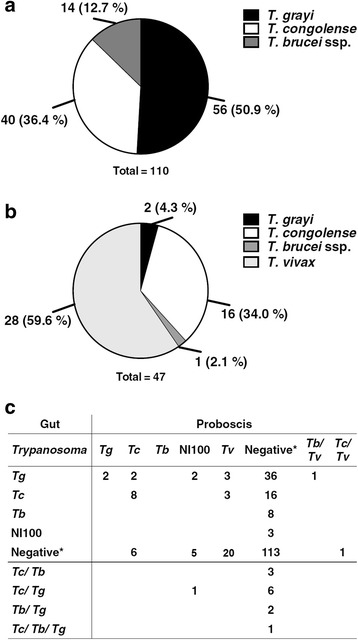
Distribution of *Trypanosoma* species in tsetse flies. **a** Relative abundance of trypanosomal DNA by species in the gut. **b** Relative abundance of trypanosomal DNA by species in proboscis. **c** Correlation of trypanosomal DNA in gut and proboscis. *Abbreviations*: *Tg*, *T. grayi*; *Tc*, *T. congolense*; *Tb*, *T. brucei* ssp.; *Tv*, *T. vivax*. If no amplicon was detected, the fly was considered to be negative

The presence of trypanosomes in gut tissue was investigated by PCR employing primers hybridising to conserved sequences in the ITS1 regions of the Kinetoplastidae DNA. Overall, 96 of 241 (40%) sampled tsetse flies were positive for trypanosomal ITS1 sequences in gut samples (Table 4 and Additional file 1: Table S1). The most prevalent *Trypanosoma* species in tsetse gut was *T. grayi* (56 flies), followed by *T. congolense* (40 flies) and *T. brucei* ssp. (14 flies) (Fig. 4a). The relative distribution of *Trypanosoma* species was similar in Dodeo and Gamba, and no significant differences were observed. *T. grayi*, *T. congolense* and *T. brucei* ssp. colonised mainly *G. p. palpalis* and *G. m. submorsitans.* The unidentified *Glossina* sp. harboured *T. grayi* and *T. congolense*. Among the 13 samples with DNA from more than one *Trypanosoma* species (Table 5), *T. congolense* occurred in 85% (11 flies), *T. grayi* in 77% (10 flies) and *T. brucei* in 46% (6 flies). The most frequent combination found was *T. congolense* with *T. grayi* (Table 5 and Additional file 1: Table S1).

**Table 4:** Distribution of the *Trypanosoma* species in *Glossina* species in the gut. Total number of *Trypanosoma* species detected

Study site	Dodeo		Alme	Gamba		Total
*Glossina/Trypanosoma*	*G. palpalis palpalis*	*Glossina* sp.	*G. morsitans submorsitans*	*G. morsitans submorsitans*	*G. tachinoides*	
*T. grayi*	26	6	1	20	3	56
*T. congolense*	27	2	0	11	0	40
*T. brucei* ssp.	9	0	0	4	1	14
Positive flies^a^	52	6	1	37	3	96
Negative flies^b^	57	12	0	69	6	145
NI100^c^	0	0	0	3	0	3

^a^Positive flies are those, in which one or more trypanosomal amplicons was detected. Therefore, the total number of positive flies is lower than the sum of *Trypanosoma* detected in a fly species at a given location

^b^If no identified amplicon was detected, the fly was considered to be negative, including presence of NI100

^c^Amplicon of 100 bp detected and not identified. The corresponding sample was recorded as NI (non identified)

**Table 5:** Distribution of the *Trypanosoma* species in *Glossina* species in the gut. Distribution of concurrent colonisation

Study site	Dodeo		Alme	Gamba		Total
*Glossina/Trypanosoma*	*G. palpalis palpalis*	*Glossina* sp.	*G. morsitans submorsitans*	*G. morsitans submorsitans*	*G. tachinoides*	
*T. congolense/brucei* ssp.	3	0	0	0	0	3
*T. congolense/grayi*	4	2	0	1	0	7
*T. brucei* ssp./*grayi*	1	0	0	0	1	2
*T. congolense/grayi/brucei* ssp.	1	0	0	0	0	1

### *Trypanosoma* species in proboscis of tsetse flies

The proboscis of tsetse flies were screened for trypanosomal DNA (Tables 6, 7 and Additional file 1: Table S2). *Trypanosoma vivax* was most prominent, followed by *T. congolense* (Fig. 4b). Surprisingly, two flies (*G. m. submorsitans*) from Gamba harboured *T. grayi* DNA.

**Table 6:** Distribution of *Trypanosoma* species in *Glossina* species in proboscis. Total number of *Trypanosoma* species detected

Study site	Dodeo		Alme	Gamba		Total
*Glossina/ Trypanosoma*	*G. palpalis palpalis*	*Glossina* sp.	*G. morsitans submorsitans*	*G. morsitans submorsitans*	*G. tachinoides*	
*T. vivax*	13	0	0	14	1	28
*T. congolense*	7	0	0	7	2	16
*T. grayi*	0	0	0	2	0	2
*T. brucei* ssp.	1	0	0	0	0	1
positive flies^a^	19	0	0	23	3	45
negative flies^b^	90	18	1	80	7	196
NI100^c^	5	2	0	0	1	8

^a^Positive flies are those, in which one or more trypanosomal amplicons was detected. Therefore, the total number of positive flies is lower than the sum of *Trypanosoma* detected in a fly species at a given location

^b^If no identified amplicon was detected, the fly was considered to be negative, including presence of NI100

^c^Amplicon of 100 bp detected and not identified. The corresponding sample was recorded as NI (non identified)

**Table 7:** Distribution of *Trypanosoma* species in *Glossina* species in proboscis. Distribution of concurrent colonisation

Study site	Dodeo		Alme	Gamba		Total
*Glossina/Trypanosoma*	*G. palpalis palpalis*	*Glossina* sp.	*G. morsitans submorsitans*	*G. morsitans submorsitans*	*G. tachinoides*	
*T. vivax/congolense*	1	0	0	0	0	1
*T. vivax*/*brucei* ssp.	1	0	0	0	0	1

*Trypanosoma vivax* and *T. congolense* colonised the proboscis of *G. p. palpalis*, *G. m. submorsitans* and *G. tachinoides*. In eight proboscis samples, NI100 was detected. Two of these samples were from *Glossina* sp., for which none of the identified trypanosomes was detected in the proboscis. Surprisingly, *T. brucei* DNA was detected in the proboscis of one *G. p. palpalis*, along with *T. vivax* DNA (Table 7 and Additional file 1).

### Concomitant colonisation of proboscis and gut by *Trypanosoma* species

*Trypanosoma vivax* was equally distributed in Dodeo and Gamba as indicated by the number of tsetse fly proboscis carrying this parasite. Similarly, the abundance of *T. grayi* colonising the gut was almost the same at both locations, whereas *T. congolense* colonising the gut were significantly (*χ* ^2^_(2)_ = 30.6, *P* < 0.0001) more frequent in flies collected in Dodeo (*G. p. palpalis*). Overall, the distribution of the trypanosomes in different tsetse fly tissues showed the expected pattern for *T. vivax* and *T. congolense* (Fig. 4). *Trypanosoma vivax* colonised the proboscis exclusively, while *T. congolense* occurred in the gut and proboscis, with the highest prevalence found in the gut (Fig. 4c). Out of the 40 flies in which *T. congolense* DNA was detected in the gut, eight flies also contained *T. congolense* DNA in the proboscis. Interestingly, *T. congolense* DNA was detected in the proboscis of additional eight tsetse flies, for which no *T. congolense* DNA was found in the gut. Also no trypanosomal DNA was detected in the gut of the tsetse fly, in which *T. brucei* ssp. DNA was found in proboscis. Surprisingly, in two of the 56 flies containing *T. grayi*, DNA from this species was also found in the proboscis (Fig. 4c). NI100 was detected in both tissues, but most frequently in proboscis (eight flies).

### *Trypanosoma* species in cattle

**Fig. 5:**
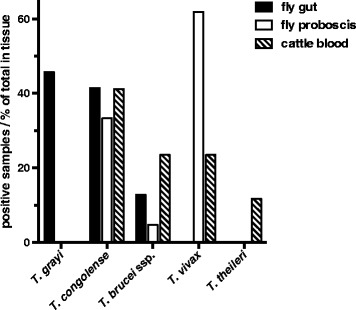
Occurrence of *Trypanosoma* species in tsetse fly gut, proboscis and cattle blood from Dodeo. Only samples from which trypanosomal DNA was amplified were included. The percentage of each *Trypanosoma* species within the different tsetse fly tissues or cattle blood is displayed

To assess trypanosomal infections of cattle in the study areas, several herds with different breeds were screened, and blood from 392 animals was collected including 120 in Dodeo, 100 in Alme, 137 in Kontcha, and 35 in Gamba (Table 8 and Additional file 1: Table S3). In Dodeo, the majority of cattle were from the Gudali breed, whereas White Fulani was more prominent in Alme, Kontcha and Gamba. In contrast to the high prevalence of trypanosomes in tsetse flies (Tables 4, 5), only a few cattle were infected with trypanosomes (Table 8), most of them in Dodeo. Furthermore, ratios between the parasite species were different in flies and cattle, as shown for Dodeo in Fig. 5.

**Table 8:** Distribution of *Trypanosoma* species in cattle sampled at indicated study sites in northern Cameroon

Study site	*Trypanosoma* species	Cattle breed			Total (*n*)	Infected (%)^a^
		Gudali (*n*)	White Fulani (*n*)	Bokolodji (*n*)		
Dodeo	*T. congolense*	7			120	14.2
	*T. brucei* ssp	3	1			
	*T. vivax*	4				
	*T. theileri*	2				
	Negative^b^	100	3			
	NI100^c^	1				
Alme	*T. theileri*		1		100	2.0
	*T. vivax*		1			
	Negative^b^	3	95			
Kontcha	*T. vivax*		1		137	1.5
	*T. grayi*		1			
	Negative^b^	32	99	4		
	NI100		1			
	Bodonidae^d^	1	2			
Gamba	*T. congolense*		1		35	5.7
	*T. theileri*			1		
	Negative^b^		24	9		

^a^Infected cattle include all animals in which DNA of an identified *Trypanosoma* sp. was detected, but not those animals in which PCR products of unknown bodonid origin were observed

^b^Negative cattle include all animals in which no identified *Trypanosoma* sp. was detected

^c^Amplicon of 100 bp detected but not identified

^d^A PCR product indicating the presence of the Bodonidae was detected

The animal pathogenic trypanosomes *T. congolense*, *T. vivax* and *T. brucei* ssp. were observed in 18/23 infections, four of these cattle were infected with *T. theileri*. An unexpected discovery was the case of *T. grayi* detected in one cow in Kontcha.

PCR products of non-identified origin were also amplified from four cattle blood samples when generic primers were used. In two of these animals, the products were similar to NI100 found in tsetse flies, whereas in the other two the products were about 250 bp. Sequencing of the latter indicated the source of DNA was derived from Bodonidae kinetoplastids, which are not parasitic in cattle [31].

### Influence of *Trypanosoma* infection on packed cell volume (PCV) of cattle blood

**Fig. 6:**
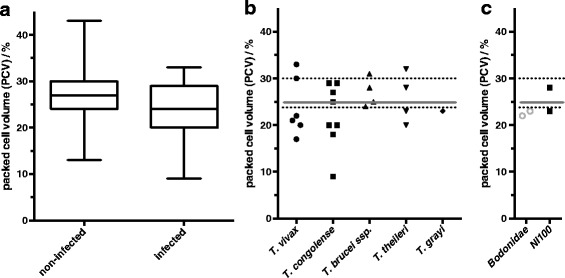
Correlation of packed cell volume (PCV) in cattle blood with the presence of Trypanosoma DNA. **a** “Non-infected” *vs* “infected” animals. Cattle are considered “infected”, if the *Trypanosoma* species as a source of a PCR product was confirmed, excluding bodonid and NI100 products. All other animals were grouped as “non-infected”. The boxes indicate the corresponding 95% confidence intervals. **b** PCVs of individual cattle, in which DNA of the indicated parasites were detected. Dotted lines indicate the 95% confidence intervals for PCV of animals, in which no trypanosomal DNA was detected; the grey line indicates the threshold PCV of 25%. **c** PCVs of individual cattle, in which DNA of Bodonidae or NI100 were detected. Dotted lines indicate the 95% confidence intervals for PCV of “non-infected” animals; the grey line indicates the threshold PCV of 25%

Anaemia has been associated with the severity of disease in animals infected with trypanosomes. Therefore, the mean PCV of infected cattle (24.1 ± 5.7%) was compared to non-infected cattle (27.1 ± 4.9%) and was found to be significantly lower (t-test: *t*_(390)_ = -2.809, *P* = 0.005) in infected animals (Fig. 6a). It should be noted that the mean PCV of non-infected animals (Table 9) from the two main breeds, Gudali and White Fulani, differed significantly (t-test: *t*_(354)_ = -2.786, *P* = 0.006). Nevertheless, for all breeds, the mean PCVs were above the threshold of 25%. The presence of several *Trypanosoma* species correlated with decreased PCVs of infected animals (Fig. 6b). The PCV was 23.0%, 23.8% ± 6.2%, and 22.1% ± 6.8%, respectively, for *T. grayi*, *T. vivax* and *T. congolense*. Interestingly, animals infected with *T. brucei* ssp. did not show a PCV below the threshold of 25%. However, two animals with *T. theileri* infection had low PCVs (20 and 23%), as was observed for those infected with bodonid kinetoplastids (23%) (Fig. 6c).

**Table 9:** Mean packed cell volume (PCV) of cattle sampled at study sites in northern Cameroon

Cattle breed	Infected cattle^a^		Non-infected cattle^b^		Prevalence (%)
	No. of samples	Mean PCV (%)	No. of samples	Mean PCV (%)	
Total	23	24.1 ± 5.7	369	27.1 ± 4.9	5.9
Gudali	16	24.8 ± 5.0	135	26.2 ± 5.1	10.6
White Fulani	6	22.5 ± 8.0	221	27.7 ± 4.6	2.6
Bokolodji	1	23	13	25.5 ± 5.8	7.1

^a^Infected cattle include all animals, in which DNA of an identified *Trypanosoma* sp. was detected, but not those animals in which PCR products of unknown or bodonid origin were observed

^b^Non-infected cattle include all animals in which no identified *Trypanosoma* sp. was detected

## Discussion

The main goal of this study was to identify the different *Trypanosoma* species colonising tsetse flies and causing trypanosomosis in cattle in Northern Cameroon. Here, we revealed the presence of several vector and parasite species in this area, including concurrent colonisation of tsetse flies with more than one *Trypanosoma* species. Our observations reveal urgent questions to be addressed regarding the infectivity and pathogenicity of various “non-pathogenic” trypanosomes.

### Distribution of *Glossina* species in the study area

Three common species of tsetse flies were identified by their morphological characteristics and *cox*1 sequences. Overall, *G. m. submorsitans* and *G. p. palpalis* were the predominant species identified. Consequently, they are likely to play a primary role in trypanosome transmission in the area. The distribution of the *Glossina* species varied in the different areas, reflecting the characteristics of the biotopes (Table 2). *Glossina m. submorsitans* dominated in the savannah of the Gamba area. Interestingly, Achukwi et al. [32] collected *G. m. submorsitans* in Guemnfiti (about 30 km north of Dodeo) and near Gamba. Their data suggested that these tsetse populations were not completely segregated. Although in that study the occurrence of trypanosomes was not analysed, it is likely that the parasites from these sites would also not be isolated populations.

*Glossina P. palpalis* was found exclusively in the Dodeo area, characterised by gallery forest. The *G. palpalis* group represent tsetse flies widely distributed in West and Central Africa [33–35]. *Glossina p. palpalis* is known to exhibit greater persistence to its location and to subsist in areas where anthropogenic developments have resulted in the disappearance of other species [36, 37].

Interestingly, in Dodeo another tsetse fly, for which no *cox*1 sequence was found in the databases, was collected, coexisting with *G. p. palpalis*. A preliminary phylogenetic analysis of *cox*1 sequences placed this species close to the *Glossina fusca* group (data not shown), which would be consistent with a recent report on the occurrence of tsetse flies identified as *G. fusca congolensis* in Dodeo by Tongue et al. [38].

In Campo, a known focus of human trypanosomiasis in South Cameroon, Farikou et al. [33] found mainly *G. p. palpalis* (94.7%) together with *G. pallicera*, *G. caliginea* and *G. nigrofusca*, which were also collected in this forestry area. While *cox*1 sequences are available for *G. pallicera* and *G. caliginea*, no such information is accessible for *G. nigrofusca* and several other *G. fusca* species at present. Therefore, it cannot be excluded that the unidentified *Glossina* sp. from Dodeo may represent one of these *G. fusca* species.

### Distribution of *Trypanosoma* species in tsetse flies

The variety of amplicons identified in this study reveals the full diversity of trypanosomes in Northern Cameroon, which hitherto was poorly understood. The overall percentage of tsetse fly gut samples positive for the presence of *Trypanosoma* was high compared to previous studies in other areas of West and Central Africa, where it ranged from 6 to 10 % [39–41]. These findings could be attributed to differences in the study sites, season and identification methods used. In this study, trypanosomal DNA was detected in 40% of tsetse fly gut and 19% of tsetse fly proboscis samples. The frequencies of concurrent colonisation by two or more *Trypanosoma* species in tsetse fly gut samples can be explained by random uptake of these most abundant parasites with the blood meals by a fly already colonised by the other *Trypanosoma* species. Therefore, the data (Tables 4, 5) do not provide evidence for competition or co-operation of these *Trypanosoma* species in gut colonisation.

In 5% of all flies, an unidentified PCR product (NI100) was obtained reproducibly. However, attempts at sequencing showed heterogeneous results and the source of these PCR products remained uncertain, and the data are not considered further.

Overall, *T. grayi* was the most prevalent trypanosome in tsetse fly gut samples (Table 4). Only a few field surveys have investigated the occurrence of *T. grayi* in tsetse flies, e.g. in the Democratic Republic of Congo [42] and the Central African Republic [43]. Originally isolated from the crocodile, *Crocodilus niloticus*, *T. grayi* has been considered a parasite of reptiles [44–47]. This seems to fit the consistent reports of this parasite in riverine species, in particular, *G. palpalis*, feeding on reptiles [41, 42]. Therefore, it was surprising to find *T. grayi* to be the most frequent trypanosome also in *G. m. submorsitans*.

To be transmitted to mammalian hosts, salivarian trypanosomes have to undergo a successive maturation process and develop into metacyclic trypomastigotes in either salivary glands or proboscis, depending on the species [8]. Our data (Fig. 4) showed a correlation between *Trypanosoma* species found in the gut and proboscis of *G. p. palpalis*, *G. m. submorsitans* and *G. tachinoides*, but not in the non-identified *Glossina* sp. collected in Dodeo. This might suggest that this *Glossina* sp. is not a good vector for *T. congolense* or *T. vivax*. However, due to the low number of flies analysed we cannot exclude *Glossina* sp. as a vector since we found only two flies with *T. congolense* in the gut and have to consider that not all gut colonisations lead to mature metacyclic trypomastigotes.

*Trypanosoma vivax* and *T. grayi* were equally distributed in Dodeo and Gamba, whereas *T. congolense* were more frequent in flies collected in Dodeo. From the 40 flies, which were colonised by *T. congolense* in the gut, eight flies were also positive in the proboscis, suggesting that the parasites had matured into the infective stage in these eight but not in the other 32 flies. Notably, in *G. p. palpalis* we observed a lower rate of *T. congolense* maturation (seven positive proboscis samples compared to 27 positive gut samples) than in *G. m. submorsitans* (seven positive proboscis samples compared to 11 positive gut samples) collected in Gamba. This observation is in agreement with a superior vector capacity of *G. m. submorsitans* for *T. congolense* than *G. p. palpalis*, as indicated in previous studies [48–50].

Strikingly, in 50% of all flies containing *T. congolense* in their proboscis, the parasite was not detected in the gut. This finding is consistent with the notion that in these cases the mid-gut colonisation by *T. congolense* has been cleared by the tsetse flies, whereas the proboscis colonisation has persisted, as has been reported previously [49, 51, 52]. Nevertheless, surveys on the prevalence of *T. congolense* trypanosomes often do not appear to take this into account, just analysing the proboscis of mid-gut-positive flies to determine infection rates. This could lead to an underestimation of the prevalence of tsetse flies carrying infective *T. congolense*.

*Trypanosoma vivax* was only detected in proboscis samples, but not in gut samples. This result is in agreement with the life-cycle of *T. vivax*, which in general only colonises mouthparts of tsetse flies, but not the gut [9], although a few studies detected *T. vivax* also in mid-gut [16, 34].

All *Glossina* species collected in this study were susceptible to colonisation with *T. grayi*. This parasite has been described to colonise the mid- and hindgut of the tsetse flies exclusively and to be transmitted via faeces [44–47]. Strikingly, in two cases we detected *T. grayi* also in the proboscis of *G. m. submorsitans*. While this could originate from a recently infected blood meal, it should be noted that in only one of these two flies a recent blood meal was observed. Based on this, it appears *T. grayi* may also be able to migrate from the gut to the mouthparts of the tsetse fly, although only at low frequency. If present in proboscis, *T. grayi* might also be transmitted via a blood meal. Even if this originates from recently infected blood meals, it would indicate that *T. grayi* might also be mechanically transmitted. This would be consistent with the detection of *T. grayi* in cattle blood. This raises the interesting question whether these trypanosomes represent a new strain with different life-cycle and host range than the ‘known’ *T. grayi*. Currently, for a phylogenetic comparison, limited sequence data are available for three different *T. grayi* subclades [46]. The comparison with the only other available ITS1 sequence (strain ANR4) revealed only a few differences, which are not sufficient for such a conclusion, especially as the number of cases is low. Further investigations need to be undertaken to investigate strain differentiation and possible transmission pathways for *T. grayi* in this region.

### *Trypanosoma* infections in cattle

The relatively high proportion of animal pathogenic trypanosomes found in tsetse flies in the present study suggests a high risk of trypanosomosis for livestock in the areas. The overall prevalence rate found in cattle was only about 6%. However, looking at the different localities the rates varied between 1.5% (Kontcha) and 14.2% (Dodeo) (Table 8), which was similar to those reported from cattle herds investigated in 2008 in the Faro division (14.3%) [18] and 2014 at several ranches in Mayo Rey (9.0%) [53]. It should be noted that in an earlier study (2001 through 2002) from sites in Faro et Deo and Vina about 40% cattle were found to be infected [11]. Although these previous studies used microscopy instead of PCR to detect the parasites, together these studies suggest a decline in trypanosomal infections in these areas over the years. This progressive decrease of trypanosomosis could be explained by an increased adoption rate of tsetse control techniques by livestock farmers. Currently, there appear to be ongoing epidemiological surveillance and updated control activities in the Adamawa region of Cameroon. These have involved screens baited with insecticides, spraying of cattle as live baits with pyrethroids (mainly acaricides) and improved use of trypanocidal drugs, which principally targets bovine trypanosomosis.

The three major known pathogenic *Trypanosoma* species found in tsetse flies were also detected in cattle, but the ratios between the parasite species were different in flies and cattle. Comparing the relative prevalence in tsetse proboscis with that in cattle blood, *T. congolense* seems to be more successfully transmitted than *T. vivax*. When analysing the situation in tsetse gut with cattle infections, *T. brucei* ssp. appears to be more successful in completing the life-cycle from gut to host blood. Since the number of infected cattle is low, inevitably conclusive correlations cannot be obtained. To investigate the transmission efficiencies of *Trypanosoma* species, an experimental study monitoring the different stages in tsetse flies and natural infection to livestock would be necessary.

Surprisingly, *T. grayi* was detected in one cow. This is remarkable since previous trials to infect mammals with *T. grayi* were not successful, as discussed by Hoare [3]. Moreover, *T. grayi* has only been found in reptiles and not from mammals [44]. At present, we cannot exclude that the *T. grayi* parasites circulating in the study area represent a strain changing host range as discussed above, in particular as it also has been detected in tsetse proboscis. To test this hypothesis, further studies are necessary to characterise *T. grayi* strains in these areas. This includes isolation of these parasites and testing Koch’s postulates for pathogens by infection experiments with mammals.

Besides the trypanosomes found in tsetse flies, *T. theileri* infections were detected in four of the 23 *Trypanosoma*-infected cattle. This observation supports the notion that non-tsetse vector(s) transmit *T. theileri*, such as the Tabanidae [54] and ticks [55]. It should be noted that the ITS1 sequences revealed that two distinct *T. theileri* lineages are circulating in the study area (Table 3). A phylogenetic study of the globally distributed *T. theileri* by Garcia et al. [29] described two main clades (I and II), which were further divided into several branches. The *T. theileri* lineages identified in this study could be assigned to clades I and IIB. Similar co-circulation of diverse *T. theileri* clades within one area had also been observed in Thailand [29].

On average, cattle with trypanosomal DNA in their blood had PCV values below a threshold of 25%. This is in agreement with previous studies [56, 57] and the notion that anaemia plays a key role in determining the severity of the infection in animals infected with trypanosomes [53]. In some cases, normal average PCV values in infected cattle were reported [58, 59], which was attributed to increased individual levels of definitive host resistance, better feeding in some cattle, or to a good body condition. It was observed here that 50% of cattle infected with *T. congolense* had PCV values below the 25% threshold. This is not surprising, as *T. congolense* is known to be the most pathogenic of these parasites in cattle. On that note, attention should be drawn to the cases of *T. grayi*, and the Bodonidae detected in this study. Their occurrence in cattle blood correlated with low PCV values similar to those observed for *T. congolense* infections. This suggests that they are potential pathogens, even though these have not been considered as pathogenic to mammals before [60]. Interestingly, animals infected with *T. theileri* parasites resembling clade IIB [29] had similarly low PCV values, whereas animals infected with *T. theileri* belonging to clade I both had normal PCV values. The observation brings up the question of changing pathogenicity of strains of parasites within one species. Obviously, it remains unknown, whether the low PCVs observed was due to the presence of these kinetoplastids or the anaemic status had other reasons, such as malnutrition or tick-borne pathogens. Along with this line, it is possible that a general poor health status promoted susceptibility to infections by these otherwise non-pathogenic parasites.

## Conclusion

The present study reveals that the diversity of trypanosomes in Northern Cameroon is more complex than previously thought. The burden of trypanosomosis could vary drastically between locations, as has been indicated by high infection rates in Dodeo compared to the other study sites. Besides the known pathogenic parasites, *T. grayi* was widely spread in the gut of tsetse flies. Unexpectedly, in a few cases, *T. grayi* appears to also colonise the mouthparts. Moreover, mammals should not be excluded as possible hosts for *T. grayi*, as it has been detected in cattle. Furthermore, it was observed that in unexpected cases *T. theileri*, *T. grayi* and bodonid infections correlated with low PCVs in cattle. These observations indicate that evolution of these parasites’ life-cycles could have caused them to become pathogenic at least in cattle with suboptimal health status. Thus, applying detailed analysis including sequencing of PCR products is necessary for monitoring the diversity of parasites, which is essential for the detection of changing pathogenicity of trypanosomes. In the case of *T. theileri*, which are transmitted by biting insects other than tsetse flies, it will be necessary to expand vector control and monitoring studies beyond tsetse flies.

## Additional files

Additional file 1: Table S1. Data for tsetse flies gut sample analysis. Sampling sites, ID code, *Glossina* species, PCR analysis with ITS1 primers (Table 1), accession numbers of sequences. Band sizes were determined by agarose gel electrophoresis and therefore, the sizes given are approximate. Based on sequences and data obtained in this study, size heterogeneity occurs for several species. The size ranges, in which we have to consider the corresponding *Trypanosoma* species are indicated in a separate column (expected). **Table S2.** Data for tsetse flies proboscis sample analysis. Sampling sites, ID code, flies species, PCR analysis with ITS1 primers (Table 1), accession numbers of sequences. Band sizes were determined by agarose gel electrophoresis and therefore, the sizes given are approximate. Based on sequences and data obtained in this study, size heterogeneity occurs for several species. The size ranges, in which we have to consider the corresponding *Trypanosoma* species are indicated in a separate column (expected). **Table S3.** Data for cattle blood sample analysis. Sampling sites, herd ID code, ID code, cattle breed, PCV, PCR analysis with ITS1 primers (Table 1), accession numbers of sequences. Band sizes were determined by agarose gel electrophoresis and therefore, the sizes given are approximate. Based on sequences and data obtained in this study, size heterogeneity occurs for several species. The size ranges, in which we have to consider the corresponding *Trypanosoma* species are indicated in a separate column (expected). **Table S4.** Nucleic acid sequences generated in this study and their accession numbers. (XLS 169 kb)

Additional file 2: Figure S1. Alignment showing the identification of *T. grayi*. The ITS1 fragment of 314 bp length was amplified with ITS1-InF and ITS1-InR from a tsetse fly gut sample (ID 237-51-00211-1-40-10, *G. tachinoides*, Additional file 1: Table S4), was subcloned, sequenced as described under Methods and the 314 bp sequence (MG234546) was then aligned against the corresponding fragment of genomic DNA from *T. grayi* ANR4 (JMRU01000589) as described in Methods. (PDF 13 kb)

## Abbreviations

BLAST: Basic Local Alignment Search Tool
*cox*1: cytochrome *c* oxidase 1
ITS1: Internal transcribed spacer 1
PCV: Packed cell volume
