# Supplementary material for: Molecular screening of tsetse flies and cattle reveal different Trypanosoma species including T. grayi and T. theileri in northern Cameroon
Source: Parasit Vectors. 2017 Dec 29;10:631. doi: 10.1186/s13071-017-2540-7 (PMC5747950; doi:10.1186/s13071-017-2540-7)
Supplement: Supplementary file 2 — Alignment showing the identification of T. grayi. The ITS1 fragment of 314 bp length was amplified with ITS1-InF and ITS1-InR from a tsetse fly gut sample (ID 237-51-00211-1-40-10, G. tachinoides, Additional file 1: Table S4), was subcloned, sequenced as described under Methods and the 314 bp sequence (MG234546) was then aligned against the corresponding fragment of genomic DNA from T. grayi ANR4 (JMRU01000589) as described in Methods. (PDF 13 kb) [file 13071_2017_2540_MOESM2_ESM.pdf]

```
MG234546        TAGAGGAAGCAAAAGTCGTAACAAGGTAGCTGTAGGTGAACCTGCAGCTGGATCATTTTC  60
                ||||||||||||||||||||||||||||||||||||||||||||||||||||||||||||
JMRU01000589    TAGAGGAAGCAAAAGTCGTAACAAGGTAGCTGTAGGTGAACCTGCAGCTGGATCATTTTC  7098

MG234546        CGATGATACTTTTCATAAAGCACACACACACACACGTGTGT---TGTGTACTCAGCGGGG  117
                |||||||||||||||| ||||||||||||||||||||||||   ||||||||||||||||
JMRU01000589    CGATGATACTTTTCATGAAGCACACACACACACACGTGTGTGTGTGTGTACTCAGCGGGG  7038

MG234546        AGAGTTCTCTTTGGGCTCTCCTTCGCACACACAGATAACACTCTTTCTCTCCACGTGTGT  177
                |||||  |||||||  |||||||||||||||||||||||||||||||| ||| |||||||
JMRU01000589    AGAGT--TCTTTGGCTTCTCCTTCGCACACACAGATAACACTCTTTCTTTCCGCGTGTGT  6980

MG234546        ATATACATATATACATACGTAGAGGGAATGAGATACCAAAAAAAGTACATGCCGCTTGAC  237
                      |||||||||||||||||||||| |||||||||||||||||||||||||||||||
JMRU01000589    ------ATATATACATACGTAGAGGGAAAGAGATACCAAAAAAAGTACATGCCGCTTGAC  6926

MG234546        TTGGCGCGTTTATTTTTTAAACGTCGCATTGTGGTTAAGCACGGCCCCAACAACGTGTCG  297
                |||||||||||| |||||||||||||||||||||||||||||||||||||||||||||||
JMRU01000589    TTGGCGCGTTTA-TTTTTAAACGTCGCATTGTGGTTAAGCACGGCCCCAACAACGTGTCG  6867

MG234546        C-ATGGATGACTTGGCTT  314
                | ||||||||||||||||
JMRU01000589    CGATGGATGACTTGGCTT  6849
```
